# Exploring microbial diversity in hot springs of Surajkund, India through 16S rRNA analysis and thermozyme characterization from endogenous isolates

**DOI:** 10.1038/s41598-023-41515-5

**Published:** 2023-08-30

**Authors:** S. Soy, U. Lakra, P. Prakash, P. Suravajhala, V. K. Nigam, S. R. Sharma, N. Bayal

**Affiliations:** 1 Department of Bioengineering and Biotechnology, Birla Institute of Technology, Mesra, Ranchi, Jharkhand 835215 India; 2 Amrita School of Biotechnology, Amrita Vishwa Vidyapeetham, Clappana, Kerala India; 3 Systems Genomics Lab, Bioclues.org, Hyderabad, India; 4 National Centre for Cell Science, Ganeshkhind, Pune, India

**Keywords:** Biotechnology, Microbiology, Molecular biology

## Abstract

Hot springs are a valuable source of biologically significant chemicals due to their high microbial diversity. To investigate the possibilities for industrial uses of these bacteria, researchers collected water and sediment samples from variety of hot springs. Our investigation employed both culture-dependent and culture-independent techniques, including 16S-based marker gene analysis of the microbiota from the hot springs of Surajkund, Jharkhand. In addition, we cultivated thermophilic isolates and screened for their ability to produce amylase, xylanase, and cellulase. After the optimized production of amylase the enzyme was partially purified and characterized using UPLC, DLS-ZP, and TGA. The retention time for the amylase was observed to be around 0.5 min. We confirmed the stability of the amylase at higher temperatures through observation of a steady thermo gravimetric profile at 400 °C. One of the thermophilic isolates obtained from the kund, demonstrated the potential to degrade lignocellulosic agricultural waste.

## Introduction

Extremophiles are microbes that survive in diverse extreme conditions such as low and high temperatures, pH, salinity, and radiation. Among extremophiles, thermophiles are bacteria that survive at elevated temperatures. They are inhabitants of various niches such as hot springs, geothermal deposits and marine steam vents^1,2^. Particularly, hot springs are unique natural environments for thermophilic microorganisms. Due to their scientific and biological significance, these thermal habitats and thermophiles have sparked research interests in recent decades. In Jharkhand, there are various hot-water ecosystems, which were formed millions of years ago by water leaching processes at the sites of radioactive mines^3^. Surajkund is the hottest hot spring in the Hazaribagh area, with temperatures ranging from 85 to 90 °C. This kund is well-known for its religious, medicinal, and aesthetic significance. Hot springs are a rich source of precious micro and macro elements, various compounds, and diverse microbial communities. The microbial community of the thermal springs is associated with the physicochemical parameters such as pH, temperature, micro and macro elements, oxido-reduction potential, and other chemical profiles^4^. Microbial diversity investigation can reveal the molecular ecological traits as well as the diversified gene pool of this natural ecosystem. However, there are no such studies on Surajkund hot springs. The microbiota of this thermal habitat is yet to be explored. In previous studies, the Surajkund sediment sample has shown the presence of novel bacterium JHK30^T^ identified as *Tepidiphilus thermophilus* and JS1^T^ identified as *Anoxybacillus*^5,6^. Also, the isolation of thermophilic bacteria at 60 °C and 70 °C with amylolytic, xylanolytic, and cellulolytic activities were reported from the same site. They were identified as *Geobacillus icigianus* and *Anoxybacillus gonensis*^7^. So far, only newly discovered bacteria or microorganisms that can secrete thermozymes have been identified in this location.

Comprehensive investigations for microbial diversity from Surajkund hot springs shall not only provide valuable information on ecological niches, but also allow for the evaluation of metabolic and functional potential of microbiota in the habitat. Also, it might help in evaluation of the molecular basis of environmental sites with distinct features and their interaction. Additionally, it helps in better understanding of how thermophilic microorganisms that survive in severe natural environments have inherent traits such as high thermostability, resistance to organic solvents, and adaptations.

Therefore, in this study, we examined the 16S rRNA data for the samples collected from five different kunds from Jharkhand, India namely Surajkund, Ramkund, Sitakund, Laxmankund, Brahmakund and one sample from a location where water from all the kunds meets i.e.Mixed kund. Though these kunds are situated in close proximity, they showed different temperatures and pH. The goal of this study is to look into microbial diversity and decipher the metabolic function of bioresources in different kunds from where samples were collected for this study.This might provide insights on the diversity of microbes present in the kunds and their industrial potential (bioactive compounds, enzymes, pigments, etc.).

## Experimental methods

### Sample collection, characterization and analysis of environmental factors

The sediment samples (water and mud) were collected from the six geographically distinct locations at the hot springs in the Surajkund dham of Jharkhand, India, viz. Surajkund hot spring, Ramkund hot spring, Laxmankund hot spring, Sitakund hot spring, Brahmakund hot spring and Mixed kund (where water from all the above mentioned kunds meet) during November 2019 (Table 1).

**Table 1:** Sampling study of hot springs of Surajkund.

Sl no.	Name of Kunds	Temperature (°C)	pH	Sample
1	Surajkund	90	7.5	Sediment
2	Laxmankund	60	8.0	Sediment
3	Brahmakund	50	8.0	Microbial mats + sediment
4	Ramkund	80	9.0	Sediment
5	Sitakund	35	7.0	sediment
6	Mixedkund	85	8.0	sediment

The sample names were as follows Surajkund, Ramkund, Laxmankund, Sitakund, Brahmakund, and Mixedkund corresponding to six different sampling locations of hot springs. The samples were collected in sterile thermo flasks bottles and transported to the laboratory in temperature control settings. Upon arrival at the laboratory, the samples were processed for bacterial DNA isolation and bacterial culture experiments. The pH and temperature of the kunds was recorded on the site of sampling and tabulated (Table 1).

### Microbial diversity analysis

#### DNA extraction

DNA extraction was carried out as described by Gothwal et al.^8^ with few modifications in the protocol. Sediment sample of 0.5 g was processed in 1 mL TENS buffer for DNA extraction. The samples were vortexed to mix thoroughly. Glass beads of 900 mg (mixture consisting of 0.1 mm, 0.5 mm and 1.0 mm diameter glass beads) were added to the sample vials. The samples were then subjected to hot SDS lysis by incubating the vials in a water bath (70 °C) for 30 min with intermittent vortexing after every 5 min. The samples were then homogenized for 5 min at maximum speed. Post homogenization, the samples were subjected to freeze thaw cycles using liquid N_2_ thrice. The samples were then centrifuged at 10,000 rpm for 10 min at room temperature and the supernatant was collected. The pellet was washed with 750 μL TENS buffer, centrifuged and supernatant was collected. The supernatant was pooled and extracted with equal volume of PCI solution. The DNA precipitation was carried out by adding 0.1 volume of 5.0 M NaCl and 2 volumes of chilled ethanol. The pellet was washed with 70% ethanol and air dried. The dried pellet was dissolved in sterile Milli-Q water and stored at – 80 °C^9^. The extracted DNA was then outsourced to a sequencing facility at Centyle Biotech Pvt. Ltd, New Delhi for Illumina MiSeq sequencing and microbial diversity analysis.

#### V3–V4 hyper-variable regions PCR amplification and sequencing

The microbial community was characterized via 16S rRNA sequencing. After the total community DNA was extracted from the sediments of six hot springs, the genomic DNA submitted for analysis was checked on 1% (w/v) Agarose gel using EtBr staining protocol. Each well was loaded with 2.0 μL of DNA sample. DNA sample quantification was performed using Qubit HS DNA quantitation kit on Qubit fluorometer. The bacterial 16S rRNA V3–V4 hypervariable was amplified using the primers V13F (5ʹAGAGTTTGATGMTGGCTCAG3ʹ) and V13R (5ʹ TTACCGCGGCMGCSGGCAC3ʹ). For polymerized chain reaction (PCR) amplification, 40 ng of extracted DNA and 10 pM of each of the primer was used with an initial denaturation at 95 °C for 15 s, annealing at 60 °C for 15 s, elongation at 72 °C for 2 min with a final extension at 72 °C for 10 min, and hold at 4 °C for 25 cycles. PCR amplification was confirmed by agarose gel electrophoresis and the amplified products were processed for further library preparation. A second round of amplification was performed to add our proprietary index sequences to the amplified products. The indexing PCR assigns barcodes to individual samples. After the second PCR, amplified products were quantified using a Qubit fluorometer and then pooled together in an equimolar concentration. The pooled products represented the 16S amplicons library, which was subjected to NGS. The concentration of the Metagenomics library was estimated using an in-house qPCR. The amplicons from each sample were purified with Ampure beads to remove unused primers and an additional 8 cycles of PCR was performed using Illumina barcoded adapters to prepare the sequencing libraries. Libraries were purified using Ampure beads and quantitated using Qubit dsDNA High Sensitivity assay kit. Sequencing was performed using Illumina Miseq^10^ with 2X300 paired-end chemistry v3 sequencing kit**.**

#### Downstream bioinformatics analysis

Standardized quality processing was applied to the raw reads of the paired-end sequence data using FastQC v0.11.2^11^ and MultiQC v1.9^12^. Prinseq v0.20.4e^13^ was used to maintain high-quality pairs with a minimum phred33 quality score of 25. Assembly of high-quality pairs was accomplished using PEAR v0.9.11^14^ with a minimum overlap threshold of 10%. The quality checked and assembled sequence data was then subjected to closed reference classification using SILVA v138.1^15^. Abundance data matrices at all five major levels of taxonomic lineage i.e., Phylum, Class, Order, Family and Genus were compiled using the OTU files of SILVA classification output. Chimera removal was done using VSEARCH pipeline. Taxonomic assessment was done for phyla and genus levels using generated OTUs^16,17^ (Table S1).

### Culture dependent studies

#### Isolation of thermotolerant and thermophilic bacteria

The soil, mud, and water samples of Surajkund and Mixedkund were processed for the enumeration and isolation of culturable moderate thermophiles (50–60 °C) and thermophiles (70 °C). The study was conducted by standard serial dilution and spread plate methodology as described by Kumar et al.^18^. Two different isolation media i.e., Nutrient Agar and Tryptone Soybean Agar/Soyabean Casein Digest Agar were employed for the isolation of thermophiles at three different high temperatures viz 50 °C, 60 °C, and 70 °C. To avoid desiccation and breaking of agar plates, the plates were covered in sterile autoclavable bags and incubated at respective high temperatures. After 24 h incubation, the agar plates were observed for different morphotypes and phenotypic characteristics.

The suitable colonies were picked on the basis of morphological difference and were continuously streaked on the same isolation media to obtain a pure culture. The obtained pure colonies were stored at 4 °C and were revived repeatedly to retain their reproducibility. The bacterial colonies were named as BITSNS, which refers to the initials of the institute’s name and the name of researchers carrying out the research.

#### Primary screening of amylase, xylanase and, cellulase producing thermophilic bacterial isolates

Primary screening was carried out using plate assay method. Respective screening medium was prepared and autoclaved at 121 °C and 15 psi for 15 min. The molten medium was poured in petri plates under aseptic conditions and pure bacterial colonies were streaked and incubated at their respective temperatures (50 °C, 60 °C, and 70 °C) for 24–48 h. Plates were observed for zone of hydrolysis.

##### Xylanolytic activity

Xylan Congo Red Agar Plate Assay^19^ was used for the identification of xylanase producing thermophilic isolates. The slight modification in the methodology was incorporation of congo red dye (0.01% w/v) in the medium. It eliminated the destain step with 1.0 M NaCl solution and thus prevented leaching out of isolates from agar surface. The purified isolates were inoculated on xylanase screening media plates and were incubated at their respective temperature for 24–48 h for the xylanase secretion. The formation of a clear zone of xylan hydrolysis indicated xylanase activity and the xylanolytic index was determined for the positive isolates as per the methodology^20^.

##### Amylolytic activity

Starch agar plate assay^21^ was used for the screening of amylase secreting isolates. The starch agar plates were spot inoculated with the pure isolates and incubated for 24–72 h for amylase secretion. After sufficient growth on plates, the plate was flooded with Gram’s Iodine and incubated for 10 min. The surplus Gram’s Iodine solution was then discarded and halo zone formation was observed which indicated starch hydrolysis and the enzymatic index (EI) for amylase activity for positive isolates were further calculated.

##### Cellulolytic activity

The screening of cellulase producing bacteria was conducted as described by Kasana et al.^22^. The isolates were spot inoculated on carboxymethylcellulose sodium salt (CMC) agar plate medium and incubated for 24–48 h. After suitable growth, cellulase enzyme expression was detected by the addition of 1 mL of Gram’s Iodine solution to the CMC plate. The plate was incubated at room temperature for 30 min and excess iodine was discarded. The isolates forming clear halo zones on CMC agar plates were selected as cellulase positive strains and the cellulolytic index was determined.

### Partial purification and molecular weight determination of amylase from *Geobacillus icigianus* (BITSNS038)

Thermophilic bacteria isolated at 70 °C, identified as *Geobacillus icigianus* showed maximum production of amylase under shake flask conditions. Partial purification of thermostable amylase from amylase producing strain BITSNS038 was carried out and was selected for further research. The precipitation of total protein was carried out using ammonium sulfate precipitation (80–95% saturation) method. Ammonium sulfate precipitation involved the slow addition of fine ground ammonium sulfate to the supernatant (crude amylase). The solution was continuously stirred slowly for 1–2 h at 4 °C. After the amount of required ammonium sulfate addition was done, the solution was kept overnight at 4 °C. The solution thus obtained was centrifuged at 10,000 rpm for 10 min, and the protein pellet obtained was dissolved in sodium phosphate buffer (pH 7.0). The resultant solution mixture was dialyzed against 10 kDa membrane^23^.

#### Ultrafiltration

The crude and concentrated amylase sample was filtered using 0.2 μm micropore syringe filter. It was further subjected to concentration using a centrifugation-based method. The membrane was rinsed by passing an ample amount of deionized water prior to ultrafiltration. Partial purification of amylase was carried out using buffer exchange with a membrane of 10 kDa (Ultra-filtration). The calibration of flow rate of retentate and permeate was also optimized using deionized water. The concentration was performed using a centrifugal concentrator of 10 kDa. The centrifugal concentrators were cleaned using 0.5 N NaOH and stored in 50% ethanol. At every step of the purification, enzyme assay and protein estimation was conducted using Nelson-Somogyi method and Bradford method respectively.

### Characterization of partially purified amylase

Characterization of partially purified amylase was performed using ultra pressure liquid chromatography (UPLC), dynamic light scattering (DLS), zeta potential (ZP), and thermogravimetric analysis (TGA), respectively.

#### UPLC analysis of partially purified amylase

UPLC (Ultra Pressure Liquid Chromatography) analysis of partially purified amylase along with the commercial amylase standard was done. The standards and sample were prepared by dissolving in deionized water with 0.1% TFA. The analysis was done at the 214 nm wavelength of the PDA detector^24^.

#### DLS-ZP analysis of partially purified amylase

DLS (dynamic light scattering) analysis was performed in order to study the dispersion of partially purified amylase in suspension. The suspension was prepared by separately mixing partially purified enzymes in deionized water (5 times dilution). The analysis was performed according to modified procedures from Jachimska et al.^25^.

ZP (zeta potential) analysis is used to determine the charge differences of the suspended particles in liquid. A value higher than − 15 mV signifies formation of colloidal particles i.e., accumulation of dissolved particles. In case of amylase, partially purified sample analysis was performed with respect to standard sample using ZP analyzer from Malvern Instruments, UK^26,27^.

#### TGA analysis of partially purified amylase

TGA (thermogravimetric analysis) of amylase was performed in order to study the thermal and oxidative stability of partially purified protein with respect to the standard. The analysis was performed using the TGA setup with a nitrogen analyzer^28^. The maximum temperature used for the analysis was 900 °C. Temporal variations in the enthalpy during the phase shifts were studied.

### Application of G. icigianus on different lignocellulosic agricultural wastes for the production of amylase

The bacteria were grown in amylase production medium supplemented with different lignocellulosic biomass (rice husk, rice straw and wheat bran) as carbon sources without any addition of starch. The composition of the amylase production medium was as follows: yeast extract 3.0 g/L, Tryptone 3.0 g/L, MgSO_4_·7H_2_O 0.2 g/L, K_2_HPO_4_ 1.0 g/L, NaCl 1.0 g/L. The pH of the medium was maintained at 7 and incubated at optimal conditions (70 °C, 150 rpm, 24 h) for amylase production. Later, the concentrations (5–12 g/L) of these agricultural substrates were also optimized for amylase production.

## Results and discussion

### Primary screening of amylolytic, xylanolytic and cellulolytic thermophilic bacteria

**Figure 1:**
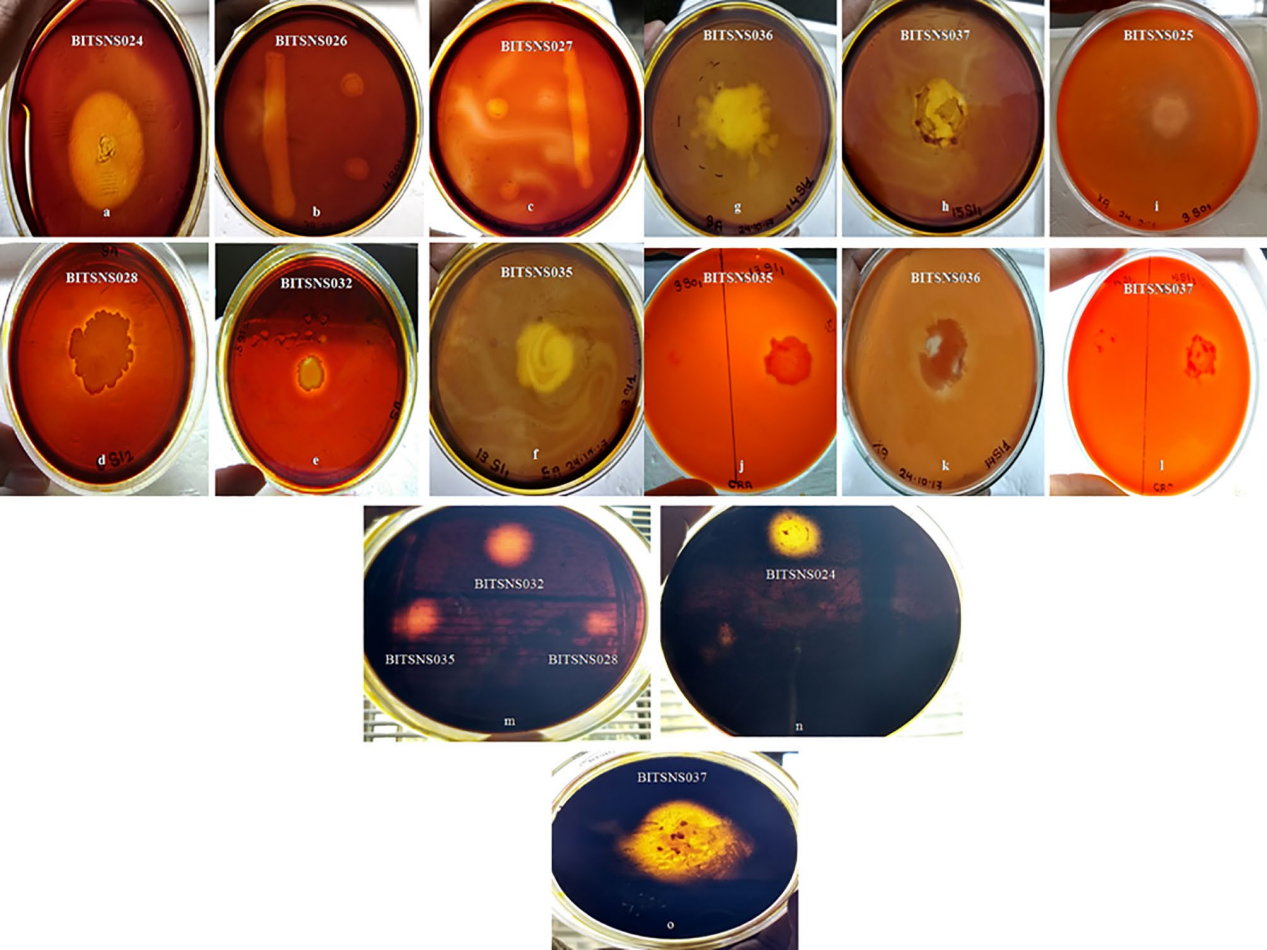
Hydrolysis zone as indicative for amylase (**a**–**h**), xylanase (**i**–**l**) and cellulase (**m**–**o**) activity screening of 60 °C isolates.

**Figure 2:**
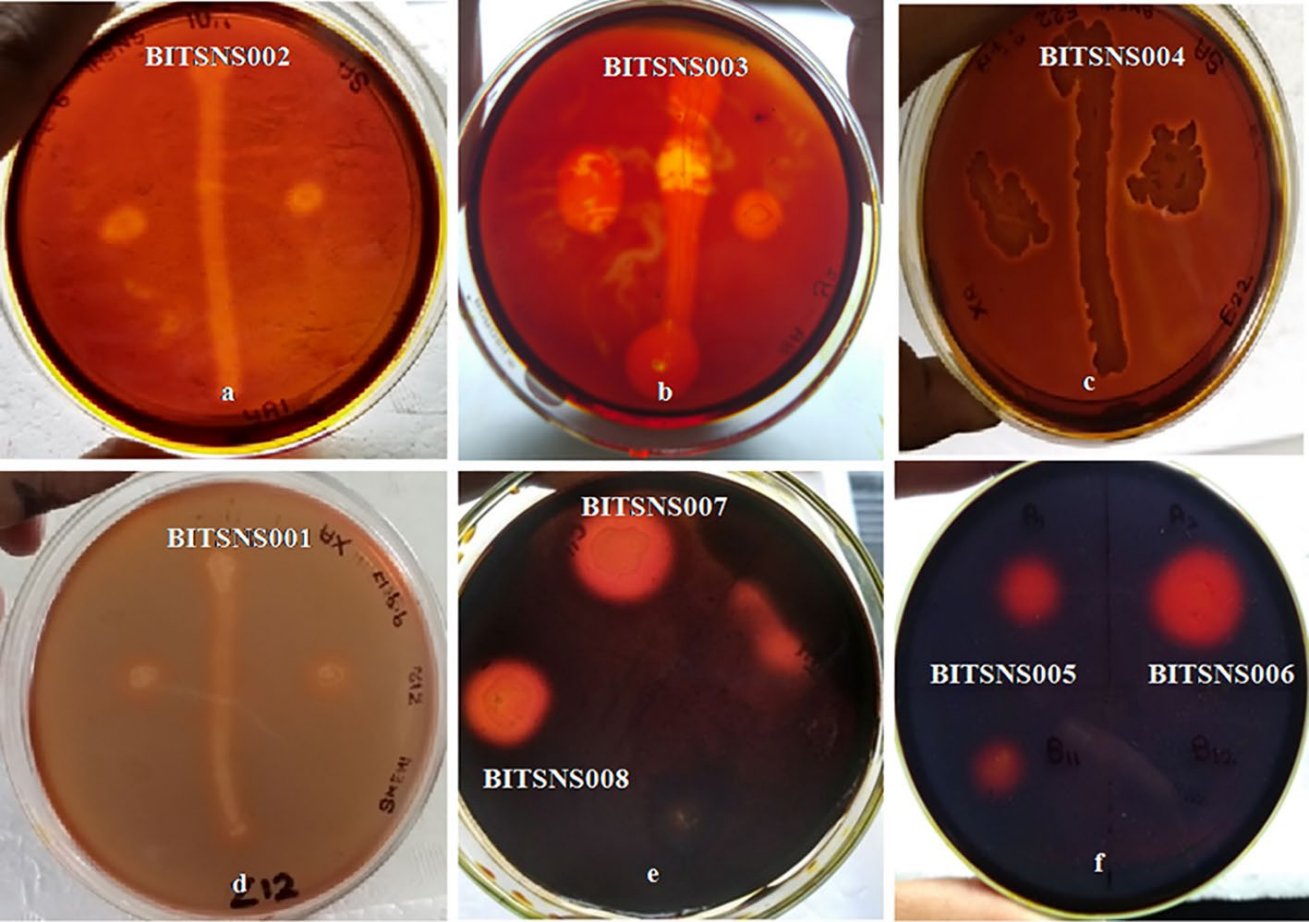
Amylolytic (**a**–**c**), xylanolytic (**d**) and cellulolytic (**e**–**f**) activity as hydrolysis zone on media plates for 50 °C isolates.

The enzyme secreting ability of 41 isolates were checked via primary screening approach on agar plate consisting of specific substrate inducer i.e. starch for amylolytic activity, xylan for xylanolytic and CMC for cellulase secreting ability. Out of 41 isolates, 23 potential enzyme secreting isolates were obtained i.e., 4 isolates of 5 °C, 15 isolates of 60 °C and 4 isolates of 70 °C having amylolytic, xylanolytic and cellulolytic enzymes secreting ability. The results of plate assay for 50 °C and 60 °C are shown in the figure (Figs. 1, 2).

The parameter for calculating the enzyme activity of isolates was the measurement of the enzymatic index (EI) by the given Eq. (1)^20^.

The enzymatic index was also calculated using formula:

1 $$EI=\frac{diameter\, of \,hydrolysis \,zone}{diameter \,of \,colony}$$

The isolates with hydrolysis zone 1.0 cm are considered significant^29^. The greater the halo zone, the higher is the EI value. Thus, higher value of EI obtained in primary screening results reflects the amylolytic, xylanolytic and cellulolytic potential of thermophiles.

### Microbial diversity of the hot springs of Suraj kund in Jharkhand, India

**Figure 3:**
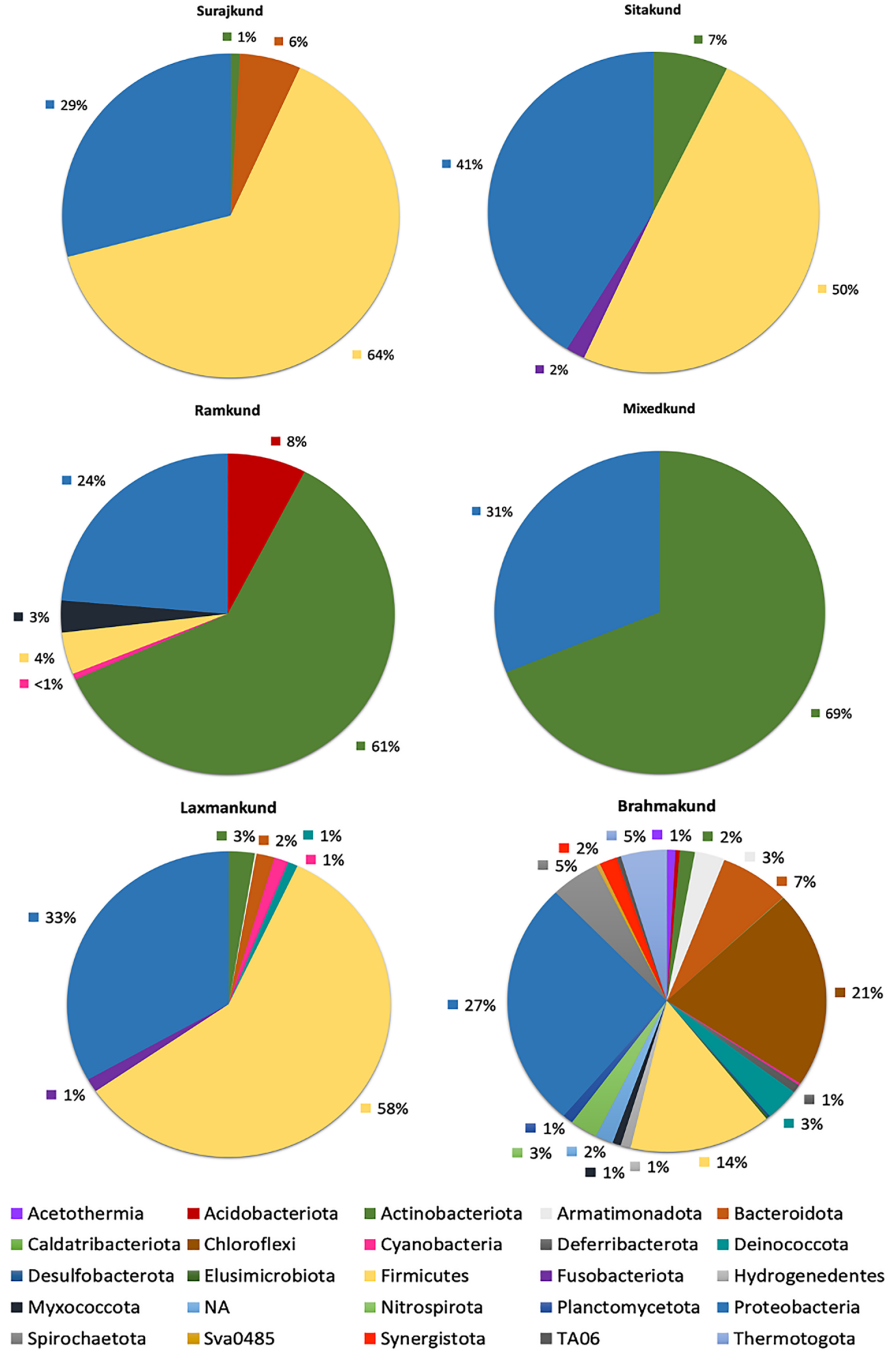
The representation of the 16S rRNA diversity of bacterial population of hot springs of Surajkund at Phylum level.

The taxonomical analysis of hot springs of Surajkund exhibited a diverse bacterial community where the presence of phylum Proteobacteria was observed in all the kunds (30.83 ± 4.17%). Phylum Firmicutes was dominant in Surajkund (64%), Laxmankund (58%) and Sitakund (50%) respectively whereas Phylum Actinobacteriota was most dominant in Mixedkund (69%) and Ramkund (61%) (Fig. 3). The study was found in accordance with another study on Surajkund by Debnath et al.^30^. In their study, it was observed that there is prevalence of the phyla Proteobacteria (> 23%) in samples from Surajkund and its surroundings apart from Chloroflexi and Deinococcus-Thermus. Temperature has always been the determining factor affecting the prevalence of Proteobacteria. Dominance of this phylum has been found in geographically distant geothermal springs like Deulajhari and Tattapani in India^31^. Proteobacteria are well known to be tolerant towards a higher concentration of sulfur and use reduced compounds of sulfur as an electron donor for carrying out their physiological processes^32^. In a study, presence of Protebacteria*,* Actinobacteria and Firmicutes in Yumthang hot spring of North Sikkim with an abundance of 54.3%, 32.2% and 6.3% respectively were reported, which indicated the presence of gram-negative bacteria in hot springs. The results also suggested that hot springs may harbour disease causing bacteria as most of the infectious bacteria are of gram-negative nature. The work also reported the abundance of thermophilic actinobacteria which holds industrial importance as a source of several enzymes such as pullulanase, amylase, DNA polymerase^33^. Proteobacteria and Firmicutes were reported in major proportion of the microbial population in Sri-Lankan Geothermal spring by Samarasinghe et al.^34^. They reported the presence of microbiota in surface water and in-depth water samples, which is lacking in the present report. While the major population of microbes in surface and in-depth water samples were different, *Klebsiella* sp. and *Pannonibacter* sp. were common. Anoxygenic photosynthetic bacteria belonging to the Rhodobacteraceae family was reported from a terrestrial hot spring in Japan. The bacteria were found to be the producer of chymotrypsin, α and β-galactosidase, trypsin and other industrially relevant enzymes. As reported in other reports the isolate was found to be gram negative and of *Alphaproteo-bacteria* class^35^. Mashzhan et al. studied Zharkent geothermal hot spring, Kazakhstan (90 °C, pH 7.5) and reported Firmicutes (89%), Proteobacteria (5.3%), and Actinobacteria (3.9%) as dominant Phylum^36^.

**Figure 4:**
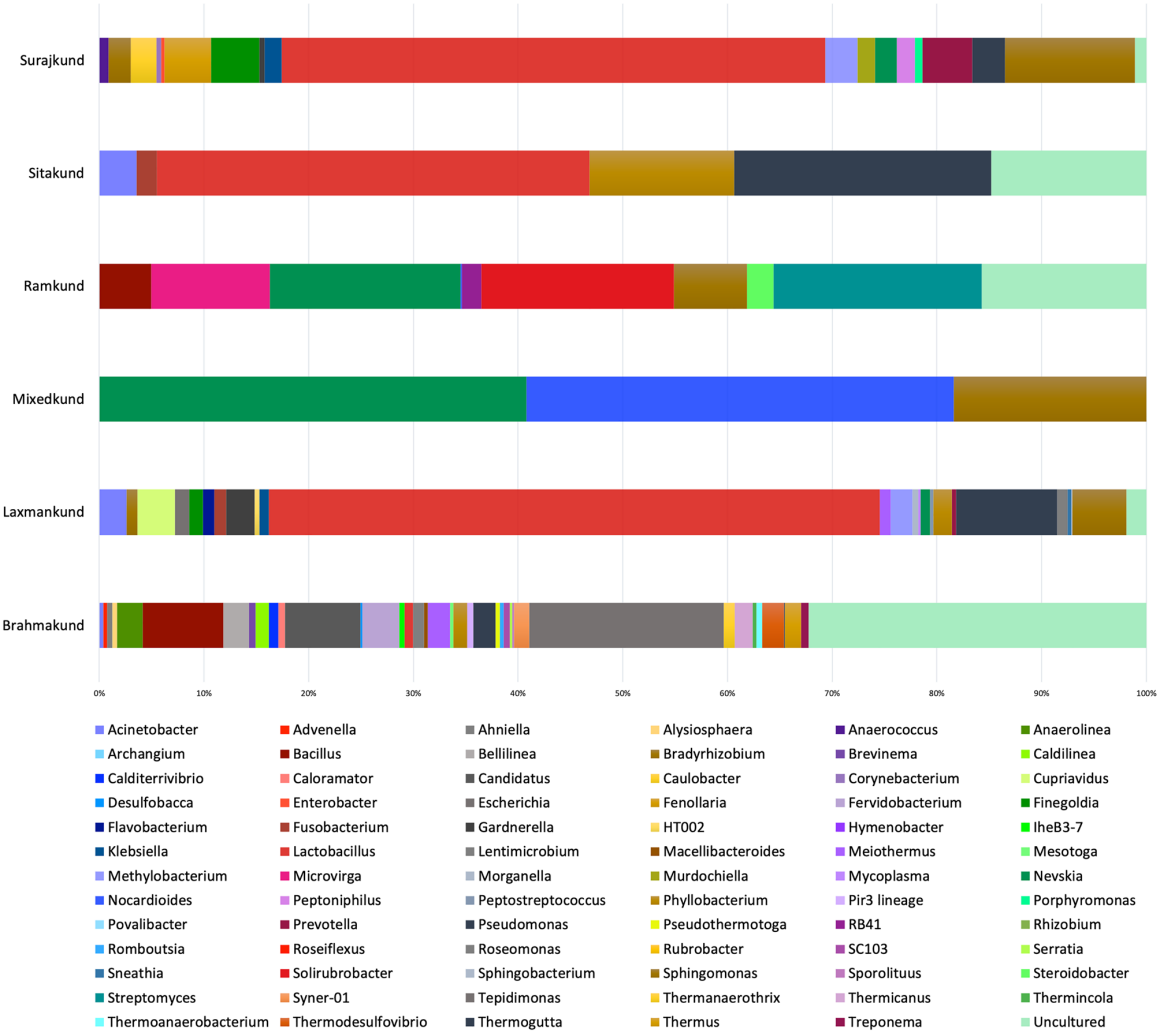
Microbial community of the hot springs of Surajkund at genus level.

In our study, phylum Chloroflexi (21%) was only detected in Brahmakund. Brahmakund situated in the vicinity of Surajkund (source) had a temperature of 50 °C and interestingly was more diverse w.r.t phyla as compared to other springs (Fig. 3). The presence of highly thermophilic phyla such as Thermatogota (5%), Deinococcota (3%), Spirochaetota (5%), Armatimondota (3%), Acetothermia (1%) as well as Desulfobacterota (1%), Nitrospirota (3%) and cyanobacteria marks an important aspect of the thermal habitat being inhabited by several microbes. At the genus level, mesophiles such as *Mesotoga*, hyperthermophilic and thermophilic genera such as *Thermodesulfovibrio*, *Thermoanaerothrix*, *Thermicanus*, *Thermincola*, *Thermogutta*, *Roseiflexus* were found to inhabit Brahmakund (Fig. 4). Also, the presence of the genus *Thermus* was astounding; as this genus is well known for its extensive usage in biotechnological applications (Table S1). Genus *Thermus* belongs to phylum Deinococcota and has been described as thermophilic Gram-negative aerobic bacteria having heterotrophic mode of nutrition. Members of this genus are commonly found in thermal springs with temperatures > 60 °C^37^.

However, the presence of *Treponema* and *Anaerococcus* as infectious genus is an alarming concern. Rest of the kunds shared a more or less similar profile at the phyla level. At the genus level, Ramkund was well dominated by *Streptomyces* (~ 20%) whereas Laxmankund and Surajkund were majorly dominated by *Lactobacillus* (~ 65%) (Table S1). Also, the presence of thermophilic genera such as *Roseomonas*, *Methylobacterium*, *Meiothermus* and, radiation resistant extremophilic genera *Solirubrobacter* and *Rubrobacter* exhibited the potential of the high temperature ecological niche supporting proliferation of extremophiles (Fig. 4).

Apart from these findings, population of the disease-causing bacteria was found in all the kunds, such as *Treponema*, *Anaerococcus*, *Mycoplasma*, *Klebsiella*, *Prevotella*, *Finegoldia*, *Fusobacterium*, and *Sphingomonas* (Fig. 4). *Sphingomonas* is known as extremely opportunistic bacteria and is well found in soil, water and hospital wastes^38^. Genera *Prevotella* is prevalent in oral, gut and vaginal microbiota^39^, while *Finegoldia* is known for serious skin infections^40^. *Fusobacterium* is known to cause acute otitis in children less than 2 years of age. When left untreated, it can lead to mastoiditis and extreme severe symptoms of bacteremia, osteomyelitis, Lemierre syndrome and septic shock^41^. The revelation in the present research is quite a matter of concern as the constant human interference and anthropogenic activities has led to the pollution of the soil and water with different kinds of wastes. The possible reason being usage of the kunds for recreational purposes and for treatment of several infectious skin diseases. The kunds with high temperature initially gives an impression that the environment might not be favorable for the proliferation of infectious microbes that usually thrive at and around 37 °C. However, the findings in the present report suggest that the change in the micro-environment due to the pollutants and the presence of the bacteria for an exceedingly longer duration has made them thermotolerant and further studies may lead to the identification of new species or strains that are thermotolerant or thermophilic in nature.

### Characterization of partially purified amylase

The amylase was partially purified having molecular weight between 45 and 63 kDa^42^. Partially purified amylase characterization was done using UPLC, DLS-ZP, and TGA.

#### UPLC analysis of partially purified amylase

UPLC analysis for the presence of amylase enzymes in partially purified fraction (Fig. S1a,b) was performed. A calibration curve of 12.5–100 µg/mL was plotted and the concentration of amylase in the precipitated and subsequently filtered ultrafiltration fraction was found to be more than 1 mg/mL. The peak of amylase was observed to be at 0.55 min. The presence of amylase was confirmed in partially purified samples by the retention time in the standard samples and the partially purified amylase enzyme. The analysis was performed only to confirm the presence of amylase enzymes.

#### DLS-ZP analysis of partially purified amylase

**Figure 5:**
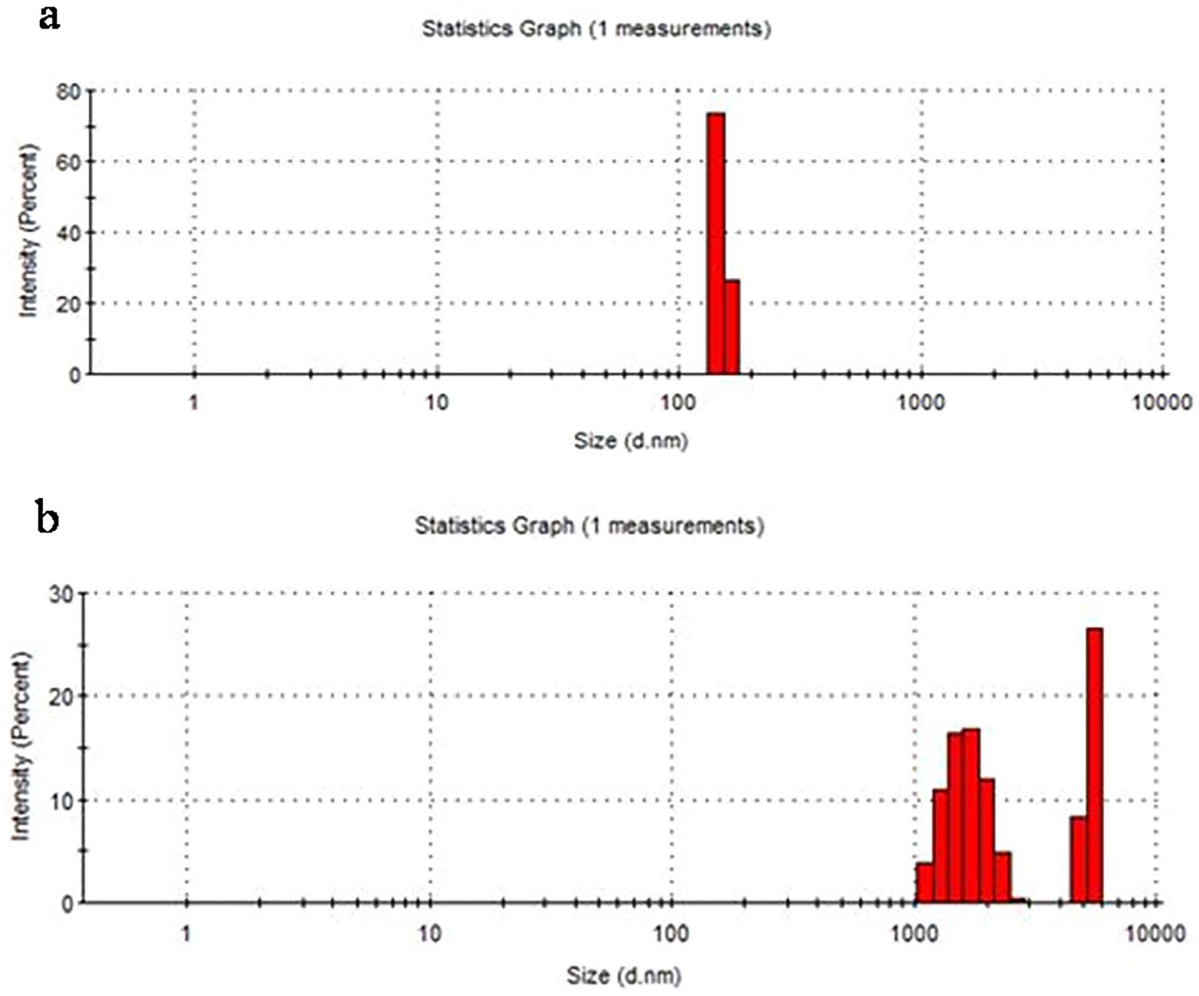
DLS analysis of (**a**) standard commercial amylase (**b**) partially purified amylase.

DLS analysis of the partially purified amylase was performed in comparison with the standard drug. It is evident from the figure (Fig. 5a,b).

The Z average was found to be less than 200 nm in the standard sample which is far less than the average particle size of partially purified amylase of around 4000 nm. In comparison to the partially purified amylase, the standard indicated higher intensity of light scattering which can be attributed to the fact that purified enzyme has reduced number of aggregates leading to the observation that standard amylase has better solubility and uniform dispersion. However, less than 100 nm of particle size has also been reported for immobilized and free amylase by Ahmad et al*.*^43^. Even smaller size of amylase segregated particles was reported (4 nm in size) by Dehnavi et al.^44^. Although the agglomerated particles had size greater than 300 nm. The particle size of less than 100 nm was also reported by Lee et al*.*^45^. The reduced size could be achieved in case of completely purified alpha-amylase.

The increase in size suggests the formation of aggregates which may be due to the partially pure nature of the enzyme. In case of a partially purified sample, zeta potential of − 12.1 mV was observed, however in comparison with the standard it was on the lower side i.e. − 21 mV. There was non-uniformity in the counts as observed in the peaks of standard amylase (Fig. S2a) and partially purified sample (Fig. S2b) indicating the nature of the enzyme to be partially purified. Weber et al*.*^46^ determined their DLS and ZP. The particle size was determined to be around 300–400 nm when the HSA (human serum albumin) was stabilized using glutaraldehyde. The zeta potential measurements of the same were found to be between − 17 and − 25 mV^47^. The purity of the sample was indicated by reduced particle size as compared to the standard.

#### Thermogravimetric analysis

TGA analysis of concentrated and partially purified amylase was performed in comparison with the commercial amylase as standard. A temperature range of 15–565 °C was kept for the standard amylase. A gradual loss was observed in the initial phase of 15–125 °C with a loss of just 3.65%. However, a rapid weight loss was observed in the next two cycles of 125–350 °C and 350–565 °C, leading to a weight loss of 99.72% (Fig. S3a).

In case of a concentrated sample, a temperature range of 30–700 °C, indicates a better stability of the concentrated enzyme at higher temperature. However, it followed the same pattern as in case of standard amylase, in terms of weight loss. A weight loss of 97% was observed indicating reduction in moisture content of the sample and confirming the concentration (Fig. S3b). The partially purified sample followed the similar pattern of weight loss when compared with the concentrated sample. However, a higher weight loss of 99.3% indicated the presence of moisture in comparison to the contaminating proteins that could have been present in the concentrated amylase (Fig. S3c). Sochava et al.^47^ reported DSC-TGA analysis of BSA (bovine serum albumin), where the maximum loss of protein occurred at a much reduced temperature of < 100 °C compared to the present work. However, in the case of *Dunaliella tertiolecta* the temperature range was broader i.e., 170–900 °C which indicated better stability of proteins extracted from microalgae^48^. Ricci et al.^49^ reported the DSCand TGA analysis of proteins purified from different legumes. The data was reported in comparison of different purity grades. It indicated that partially purified (medium purity) enzymes are more stable at higher temperatures as compared to the completely purified enzymes. This is indicative of the need of micro-environments for better stability; however this was not true for the protein isolated from all the sources. Total protein isolated from beans had nearly no effect on the temperature stability while better stability in medium purified samples was found in case of protein isolated from lentils. In case of peas the stability was found to be more in 68% purified sample compared to 77% purified sample**.**

#### Application of G. icigianus on different lignocellulosic agricultural wastes for the production of amylase

The use of low-cost agriculture wastes as substrates for the production of industrial enzymes is an economical and significant way to reduce the cost of the overall process. *G. icigianus* (strain BITSNS038) was able to utilize a variety of inexpensive substrates such as wheat bran, rice husk, and rice straw for production of amylase (Fig. S4).

Interestingly, all lignocellulosic substrates supported amylase production by *G. icigianus*. The amylase activity was maximum for rice husk (77.5 U/mL at 10 g/L) followed by wheat bran (55.27 U/mL at 10 g/L). Minimum activity was observed for rice straw with 1.52 U/mL (result not shown). The low activity compared to starch is due to presence of cellulose, hemicellulose, lignin and silica etc. in the lignocellulosic substrates. *Geobacillus* sp. has versatile catabolic activity, rapid growth rates, and is known for the degradation of hemicelluloses and starch^50^. These intriguing characteristics make it a potential candidate in second-generation (lignocellulosic) biorefineries for biofuel production. Agricultural wastes have a significant amount of lignocellulosic carbohydrate fraction that can be hydrolyzed and fermented for the bio-fuel production^51^. The maximum amylase production from rice husk and wheat bran is attributed to their rich profile which consists of high carbon, nitrogen, cellulose and hemicelluloses. Rice straw on the other hand has some challenges such as high inorganic composition and C/N ratio that affects its biodegrability^52^.

## Conclusion

Surajkund and other neighbouring hot springs in Jharkhand which we have explored for thermophilic microbiota may be used as a source of thermostable enzymes of industrial importance. Our previous studies have proved that *Anoxybacillus gonensis* and *Geobacillus icigianus,* isolated from the hot spring of Surajkund have the ability to secrete thermostable enzymes^7,42^. The diversity of microbiome in these hotsprings of Jharkhand through 16S rRNA analysis revealed that Proteobacteria was observed in all the kunds, phylum Chloroflexi (21%) was only detected in Brahmakund. Partially purified amylase from *G. icigianus* from Surajkund has been characterized in terms of its purity, thermostability as well as its ability to biodegrade lignocellulosoic agricultural wastes. The presence of *Thermus* genus, and radiation resistant bacteria *Rubrobacter* also reveals the potential of the hot springs as reservoir of industrially important microbes.

### Supplementary Information

Supplementary Figures.

Supplementary Tables.

## Data Availability

The data supporting the findings of this study are available in NCBI-GenBank with BioProject ID PRJNA940280.

## Abbreviations

EI: Enzymatic index
CMC: Carboxymethylcellulose sodium salt
UPLC: Ultra pressure liquid chromatography
DLS: Dynamic light scattering
ZP: Zeta potential
TGA: Thermogravimetric analysis
C/N ratio: Carbon to nitrogen ratio
