# Supplementary material for: Exploring microbial diversity in hot springs of Surajkund, India through 16S rRNA analysis and thermozyme characterization from endogenous isolates
Source: Sci Rep. 2023 Aug 30;13:14221. doi: 10.1038/s41598-023-41515-5 (PMC10469164; doi:10.1038/s41598-023-41515-5)
Supplement: Supplementary file 1 — Supplementary Figures. [file 41598_2023_41515_MOESM1_ESM.doc]

**Article Title:**

**Exploring Microbial Diversity in Hot Springs of Surajkund, India through 16S rRNA Analysis and Thermozyme Characterization from Endogenous Isolates**

**Journal Name:** Scientific Reports

**^1^S. Soy, ^1^U. Lakra, ^1^P. Prakash, ^2^P. Suravajhala, ^1^V.K. Nigam, ^1^S. R. Sharma*^3^, N.Bayal**

**^1^Department of Bioengineering and Biotechnology, Birla Institute of Technology, Mesra, Ranchi, India**

**^2^Amrita School of Biotechnology, Amrita Vishwa Vidyapeetham, Clappana, Kerala, India**

**^3^National Centre for Cell Science, Ganeshkhind, Pune, India**

***Corresponding author:** [**srsharma@bitmesra.ac.in**](mailto:srsharma@bitmesra.ac.in)

Postal address: Dr. S. R. Sharma, Assistant Professor

Department of Bioengineering and Biotechnology,

Birla Institute of Technology, Mesra

Ranchi, Jharkhand, India-835215

ORCID:0000-0002-9201-3532

**
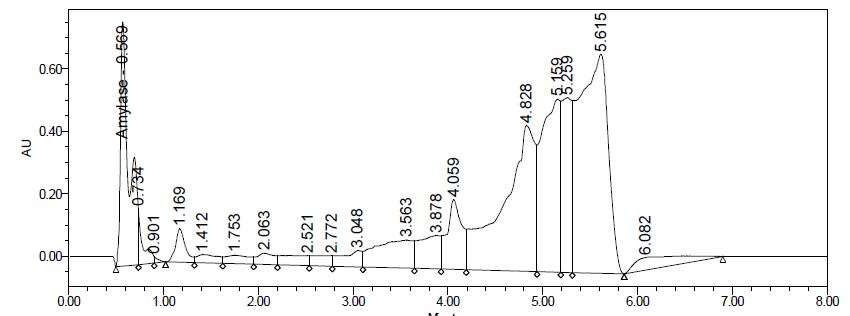

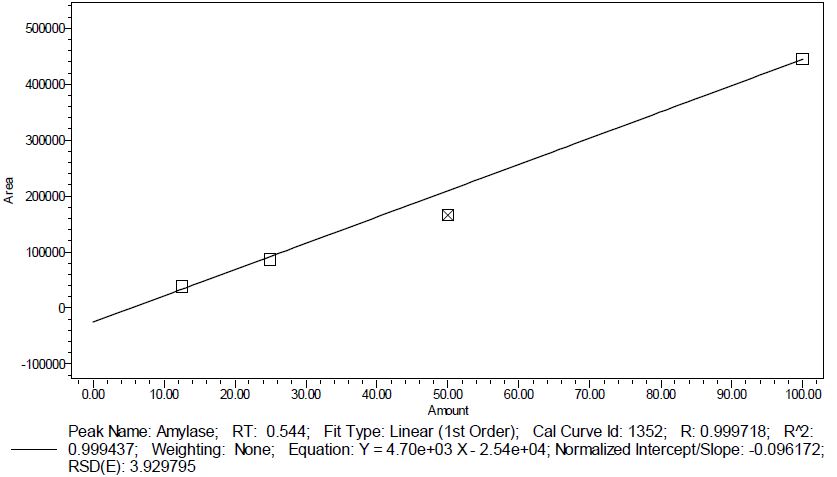
**

**b.**

**a.**

**Fig. S1.** (a) UPLC chromatogram of partially purified amylase with retention time at 0.569 minutes (b) Calibration plot of UPLC with the range of standard amylase (12.5 to 100 µg/mL)

**
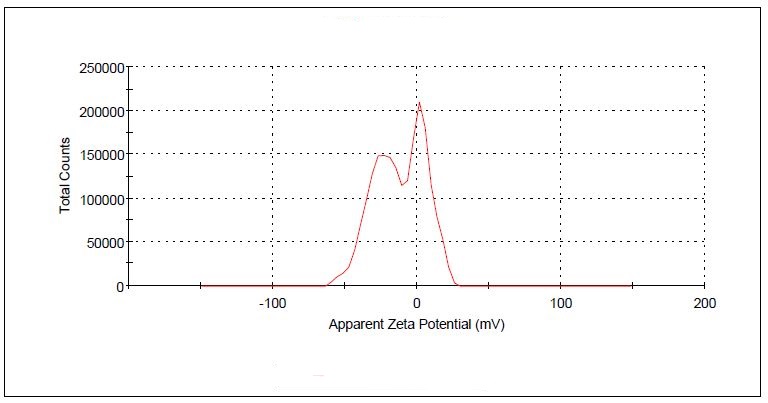

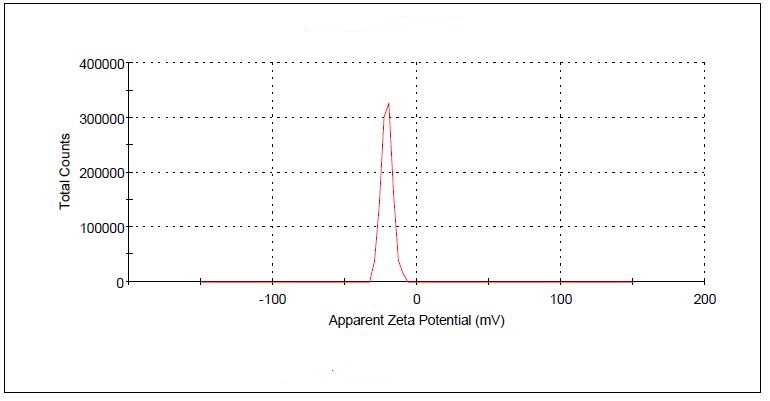
Fig. S2.** ZP analysis of (a) standard commercial amylase (b) partially purified amylase

**a.**

**b.**

**aa...**

**b**

**c**

**Fig. S3.** TGA profile of (a) standard protein (b) concentrated protein (c) partially purified protein


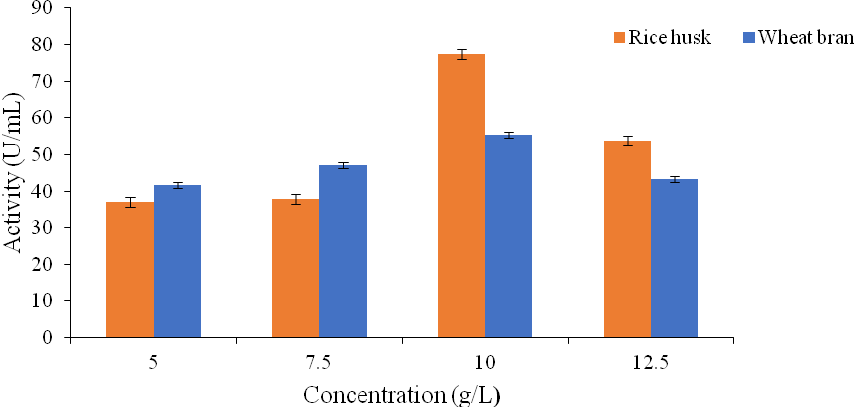


**Fig. S4.** Amylase production from agricultural wastes

**List of Supplementary Tables:**

**Supplementary Table S1:** Operational Taxonomic Unit (OTU) file for the taxonomic assessment of microbiota of hot springs of Surajkund
